# Change in post‐task resting state EEG differences between cognitively normal adults with and without elevated PET Amyloid

**DOI:** 10.1002/alz70861_107930

**Published:** 2025-12-23

**Authors:** Amanda Cook Maher, Jordan R Bross, Carol C Persad, McKenna Sawitz‐Warren, Bruno Giordani, Voyko Kavcic

**Affiliations:** ^1^ Michigan Alzheimer's Disease Research Center, Ann Arbor, MI USA; ^2^ University of Michigan, Ann Arbor, MI USA; ^3^ Wayne State University, Detroit, MI USA

## Abstract

**Background:**

Accurate identification of individuals at risk for Alzheimer’s disease (AD) is critical for early intervention. Elevated brain amyloid (Ab) is an early AD risk biomarker, however, PET imaging is expensive and not widely available. Prior work using lower‐cost EEG spectral power demonstrates a slower return to resting state following task completion (rsEEG‐TA effect) for individuals with amnestic Mild Cognitive Impairment compared to cognitively normal adults. This pilot evaluates the rsEEG‐TA effect earlier in the AD course by studying cognitively normal older adults with and without elevated Ab.

**Method:**

Data included 29 cognitively normal older adults with an amyloid‐PET scan and EEG before and after a cognitively complex, ecologically valid simulated driving task. Nine participants were Ab‐positive (≥20 centiloids) and 20 were Ab‐negative (≤10 centiloids). Eyes closed EEG data was collected using a dry 30‐channel and processed offline. Fast Fourier transforms evaluated spectral power in four major frequency bands and within four brain regions of interest (frontal, central, parietal, occipital). General linear models were used to analyze potential Ab‐group differences in rsEEG‐TA as a percent change in rsEEG spectral power within each brain region and separately for each frequency band.

**Result:**

There were no demographic group differences. Pre‐task, there was a significant effect of brain region on rsEEG spectral power, but no amyloid group or interaction effect. A significant effect of Ab group on rsEEG‐TA was seen within delta (*p* =0.013) with a trend toward a brain region by Ab status interaction (*p* =0.060). Overall, Ab‐positive participants had a larger negative percent change, suggesting a slower post‐task return to pre‐task resting state, versus Ab‐negative participants. No significant effects were seen across other frequency bands.

**Conclusion:**

While Ab‐positive and ‐negative cognitively normal adults appeared similar before completing a complex driving simulator task, Ab‐positive adults had a greater decline in rsEEG spectral power following task completion. Thus, the rsEEG‐TA effect may be evident even before overt cognitive changes are detected. Given the association of elevated delta power in AD, additional investigation is warranted as to whether post‐task slowing in the return to rsEEG within delta is an early marker of amyloid status and AD.

